# The efficacy of infliximab combined with partial enteral nutrition in the treatment of Crohn’s disease: a cohort study

**DOI:** 10.3389/fnut.2025.1591954

**Published:** 2025-06-18

**Authors:** Chen Huang, Chao Chen, Hao Wu, Hanyu Yin, Weixiang Yao, Susu Bai, Baixue Zhuo, Xiaoli Wu

**Affiliations:** ^1^Department of Gastroenterology, The First Affiliated Hospital of Wenzhou Medical University, Wenzhou, China; ^2^Department of Gastroenterology, Wencheng County People's Hospital, Wenzhou, China; ^3^Department of Gastroenterology, Taishun People's Hospital, Wenzhou, China

**Keywords:** Crohn’s disease, infliximab, partial enteral nutrition, clinical response, endoscopic remission, safety

## Abstract

**Background and aims:**

The issue of loss of efficacy with infliximab (IFX) treatment in Crohn’s disease (CD) significantly limits its clinical use. This study aims to investigate the role of therapy combined with partial enteral nutrition (PEN) in maintaining the efficacy of infliximab.

**Methods:**

Consecutive CD patients undergoing IFX for induction and maintenance therapy were included, with a follow-up period of at least 54 weeks and endoscopy performed around 54 weeks. Subsequent longitudinal monitoring evaluated improvements in the Crohn’s Disease Activity Index (CDAI) score at 14 weeks and endoscopic remission at 54 weeks.

**Results:**

Among the 176 included patients, 99 (56%) were in the IFX monotherapy group, and 77 (44%) were in the IFX + PEN group. A significantly higher proportion of patients in the IFX + PEN group achieved clinical response (defined as a CDAI decrease ≥70 points) compared to those in the IFX group at 14 weeks (87.01% vs. 74.75%, *p* = 0.043), as well as a higher proportion achieving endoscopic remission at 54 weeks (84.42% vs. 65.66%, *p* = 0.005). Meanwhile, combination therapy with PEN emerged as an independent protective predictor of endoscopic remission at 54 weeks in two multivariate-adjusted models, with ORs of 3.34 and 3.33, respectively (both *p* < 0.05). Subgroup analysis and interaction test results further supported that all CD patients can benefit from combination therapy with PEN.

**Conclusion:**

Infliximab treatment combined with partial enteral nutrition is beneficial for both short-term clinical response and long-term endoscopic remission in CD patients.

## 1 Introduction

Crohn’s disease (CD) is a chronic inflammatory disease of the gastrointestinal tract, characterized by recurrent flares and progressive tract damage (1). Over the past several decades, there has been an increase in the incidence and prevalence of CD worldwide, particularly in newly industrialized countries (2). Currently, CD remains incurable with no clear etiology. Therefore, the primary treatment strategies focus on early control of inflammation to achieve remission, progressing from clinical remission to endoscopic remission (3).

Since the advent of infliximab (IFX) at the turn of the century, the era of biologics has significantly altered the natural history of CD (4). Compared with conventional treatments such as corticosteroids and immunomodulators, biologics not only demonstrate dramatic efficacy but also carry a lower risk of adverse effects (5). Currently, biologics are widely used for the induction and maintenance treatment of patients with moderate to severe CD after the failure of conventional therapies and are recommended as first-line biological treatment by authoritative guidelines worldwide (6, 7). However, the loss of response (LOR) or efficacy to biologics treatment has become a significant challenge for clinicians in real-world practice. Several studies have indicated that approximately 10–40% of patients do not respond to induction therapy, and 23–46% of patients experience loss of efficacy over time (8–10). Even Gisbert and Panés (11) estimated that the annual risk of flare is 13% per patient-year of IFX treatment. Combination therapy with immunomodulators, dose escalation, or switching to another biologic are considered potential countermeasures; However, associated disadvantages such as safety concerns and economic burdens also warrant attention (12–15). Therefore, reducing the risk of treatment failure while minimizing additional burdens is of significant clinical importance.

The effect of nutritional therapy for CD patients is increasingly gaining attention from researchers. In pediatric CD patients, nutritional therapy, compared with corticosteroids, has been proven to be a more effective approach for induction therapy, with a lower risk of adverse prognostic events (16–18). Literature also indicates that CD patients with poor nutritional status are associated with unfavorable clinical outcomes, such as increased frequency of flares and reduced response to medical therapy (19, 20). Recently, a meta-analysis suggested that anti-tumor necrosis factor-*α* antibodies (anti-TNF-α) therapy combined with enteral nutrition (EN) is effective in maintaining remission. However, it is noteworthy that different studies did not adopt consistent definitions of disease flares or LOR to IFX as primary endpoints, and the patient populations involved were highly heterogeneous (21).

Nutritional therapy has no significant toxic side effects and can effectively avoid adverse effects caused by interactions between concomitant medications. Additionally, it also possesses potential therapeutic value (21). For these reasons, we conducted this study to investigate whether IFX treatment combined with EN contributes to maintaining IFX efficacy.

## 2 Materials and methods

### 2.1 Study design

This is a single-center, retrospective cohort study, and the entire study will continue until January 2025. The study was conducted in accordance with the Declaration of Helsinki and was approved by the Ethics Committee of the First Affiliated Hospital of Wenzhou Medical University, with the need for written informed consent waived.

### 2.2 Patients enrollment

This study enrolled consecutive patients at The First Affiliated Hospital of Wenzhou Medical University from January 2012 to January 2024. All these patients met the following criteria. Inclusion criteria: (1) Diagnosed with Crohn’s disease according to the Chinese Crohn’s Disease Diagnosis and Treatment Guidelines (2023, Guangzhou); (2) Underwent infliximab for induction and maintenance therapy; (3) Completed at least 54 weeks of follow-up and underwent endoscopy at around 54 weeks; Exclusion criteria: (1) Unclassified CD; (2) Lack of detailed or incomplete medical records; (3) Required nutrient supplementation solely through parenteral nutrition or total enteral nutrition; (4) Had coexisting conditions that could potentially influence results, such as hematological system-related diseases, malignancies, active tuberculosis, viral hepatitis, autoimmune diseases, or acquired or congenital immunodeficiency.

### 2.3 Treatment and group

All involved patients received IFX treatment. During the induction phase of IFX treatment, 5 mg/kg of the drug was administered at weeks 0, 2, and 6. Subsequently, as maintenance therapy, 5 mg/kg of IFX was administered every 8 weeks.

For CD patients, the daily energy requirement was calculated as 30–35 kcal·kg^−1^·d^−1^. Patients who obtained at least half of their energy requirement from enteral nutrition therapy were assigned to the infliximab combined with partial enteral nutrition (IFX + PEN) group, while the others were assigned to the infliximab monotherapy (IFX) group.

### 2.4 Date collection and evaluations of treatment

We collected the following clinical information from the patients’ medical records: age, sex, disease duration, Montreal classification, history of previous CD-related surgery, history of treatment with biologics, concomitant immunomodulatory treatment, blood test results, endoscopy results, body mass index (BMI), calculated Crohn’s Disease Activity Index (CDAI) score, records of enteral nutrition use, and adverse events. The Montreal classification serves as the international standard for Crohn’s disease subtyping, categorizing age at diagnosis (A1: <16 years, A2: 17–40 years, A3: >40 years), disease location (L1: ileal, L2: colonic, L3: ileocolonic, L4: upper digestive tract involved), and disease behavior (B1: non-structuring and penetrating, B2: structuring, B3: penetrating) throughout the disease course.

The primary endpoint of this study was the rate of maintaining remission at 54 weeks. Endoscopic remission was defined as Simple Endoscopic Score for Crohn’s Disease (SES-CD) < 3 with no ulcer and recurrence was defined as SES-CD ≥ 3 with or without ulcers. During 54-week follow-up period, patients who required additional concomitant therapy, dose escalation (including a dose increase or a shortened interval of IFX), surgery, and hospitalization due to exacerbated CD were also classified as recurrence. Shortened interval of IFX was defined as a duration of 4 weeks or less. The secondary endpoints were rate of clinical response at 14 weeks. Clinical response was defined as a CDAI change (ΔCDAI) ≥ 70-point decrease from the baseline (week 0) CDAI score. Other objectives, such as clinical remission (defined as CDAI <150), were also evaluated.

### 2.5 Safety

Adverse events, including serious infections, infusion-related reactions, hematologic conditions, hepatic insufficiency, congestive heart failure, demyelinating neurological disorders, and CD-related unexpected hospitalization, were also recorded during the 54-week follow-up.

### 2.6 Statistical analysis

Continuous variables are expressed as mean ± standard deviation (SD) or median (interquartile range [IQR]: first quartile and third quartile). Differences between two groups were compared using independent two-sample Student’s *t*-tests for normally distributed data or Mann–Whitney U tests for non-normally distributed data. Within-group comparisons were assessed using the Wilcoxon signed-rank test. Categorical variables are presented as numbers (percentages), and differences were examined using chi-square or Fisher’s exact tests, as appropriate. Univariate logistic regression analyses were performed to estimate the odds ratios (ORs) for achieving endoscopic remission based on various baseline characteristics. Multivariate logistic regression models were constructed with endoscopic remission as the dependent variable. Variables that altered the estimates of the effect of combination therapy with PEN on endoscopic remission by more than 10% or had *p* < 0.10 in the univariate logistic analyses were included as potential confounders in the final models. Subgroup analyses were conducted to evaluate whether potential covariates (age, sex, BMI, disease location, disease behavior, and CD-related surgical history) modified the relationship between combination therapy with PEN and endoscopic remission at 54 weeks. Interactions were assessed using the likelihood ratio test. Data management and analyses were performed using SPSS version 20.0 (SPSS, Chicago, Illinois), EmpowerStats^1^ and the statistical package R. All tests were two-sided, and a *p*-value <0.05 was considered statistically significant.

## 3 Results

### 3.1 Baseline patient characteristics

A total of 176 patients participated in this study, and their baseline characteristics are detailed in Table 1. 56% received IFX monotherapy, and 44% received IFX combined with PEN. Significant differences were observed in disease behavior and history of previous CD-related surgery, while the distribution of age, gender, age at diagnosis, disease location, history of previous biologic use, and concomitant immunomodulatory treatment did not significantly differ between the two groups. Patients in the IFX + PEN group exhibited a lower BMI compared to those in the IFX group (18.45 ± 2.31 kg/m^2^ vs. 19.84 ± 3.05 kg/m^2^, *p* = 0.001). Conversely, the CDAI scores were significantly higher in the IFX + PEN group (285.34 ± 98.80 vs. 242.95 ± 101.49, *p* = 0.006). Laboratory parameters, including C-reactive protein (CRP) [18.10 (9.55–40.45) mg/L vs. 16.40 (5.26–34.45) mg/L, *p* = 0.161] and albumin (ALB) levels (38.19 ± 5.55 g/L vs. 37.23 ± 5.46 g/L, *p* = 0.255), did not differ significantly between the two groups.

**Table 1:** Baseline characteristics of study cohorts.

Variable	Total (*n* = 176)	IFX group (*n* = 99)	IFX + PEN group (*n* = 77)	*p*-value
Age (years)	26.38 ± 9.34	26.59 ± 9.54	26.10 ± 9.12	0.735
Disease duration (months)	2.00 (1.00–28.75)	2.00 (1.00–22.50)	3.00 (1.00–32.00)	0.442
BMI (kg/m^2^)	19.23 ± 2.83	19.84 ± 3.05	18.45 ± 2.31	0.001
CDAI score	261.50 ± 102.24	242.95 ± 101.49	285.34 ± 98.80	0.006
SES-CD	9.00 (6.00–15.00)	9.00 (5.00–15.00)	9.00 (6.00–15.00)	0.646
CRP (mg/L)	18.00 (7.51–38.40)	18.10 (9.55–40.45)	16.40 (5.26–34.45)	0.161
ALB (g/L)	37.77 ± 5.52	38.19 ± 5.55	37.23 ± 5.46	0.255
Sex				0.150
Female, *n* (%)	39 (22.16%)	18 (18.18%)	21 (27.27%)	
Male, *n* (%)	137 (77.84%)	81 (81.82%)	56 (72.73%)	
Age at diagnosis, *n* (%)				0.460
A1 (≤16 years), *n* (%)	23 (13.07%)	11 (11.11%)	12 (15.58%)	
A2 (17–40 years), *n* (%)	140 (79.55%)	79 (79.80%)	61 (79.22%)	
A3 (>40 years), *n* (%)	13 (7.39%)	9 (9.09%)	4 (5.19%)	
Disease location				0.874
L1 (ileal), *n* (%)	42 (23.86%)	23 (23.23%)	19 (24.68%)	
L2 (colonic), *n* (%)	32 (18.18%)	17 (17.17%)	15 (19.48%)	
L3 (ileocolonic), *n* (%)	102 (57.95%)	59 (59.60%)	43 (55.84%)	
L4 (upper digestive tract involved) (yes/no), *n* (%)	16 (9.09%)/160 (90.91%)	12 (12.12%)/87 (87.88%)	4 (5.19%)/73 (94.81%)	0.113
Disease behavior				0.017
Structuring and penetrating, *n* (%)	5 (2.84%)	0 (0.00%)	5 (6.49%)	
B1 (non-structuring and penetrating), *n* (%)	112 (63.64%)	70 (70.71%)	42 (54.55%)	
B2 (structuring), *n* (%)	46 (26.14%)	24 (24.24%)	22 (28.57%)	
B3 (penetrating), *n* (%)	13 (7.39%)	5 (5.05%)	8 (10.39%)	
Perianal disease (yes/no), *n* (%)	82 (46.59%)/94 (53.41%)	48 (48.48%)/51 (51.52%)	34(44.16%)/43 (55.84%)	0.568
Extra-intestinal manifestations (yes/no), *n* (%)	8 (4.55%)/168 (95.45%)	6 (6.06%)/93 (93.94%)	2 (2.60%)/75 (97.40%)	0.274
Smoking status (yes/no), *n* (%)	14 (7.95%)/162 (92.05%)	10 (10.10%)/89 (89.90%)	4 (5.19%)/73 (94.81%)	0.233
CD-related surgical history (yes/no), *n* (%)	22 (12.50%)/154 (87.50%)	7 (7.07%)/92 (92.93%)	15 (19.48%)/62 (80.52%)	0.014
Concomitant immunomodulatory treatment (yes/no), *n* (%)	48 (27.27%)/128 (72.73%)	29 (29.29%)/70 (70.71%)	19 (24.68%)/58 (75.32%)	0.495
Use of biologics for the first time (yes/no), *n* (%)	149 (84.66%)/27 (15.34%)	85 (85.86%)/14 (14.14%)	64 (83.12%)/13 (16.88%)	0.617

BMI, body mass index; CDAI score, Crohn’s Disease Activity Index score; SES-CD, Simple Endoscopic Score for Crohn’s Disease; CRP, C-reactive protein; ALB, Albumin.

### 3.2 Efficacy evaluation at 14 weeks and 54 weeks

**Figure 1:**
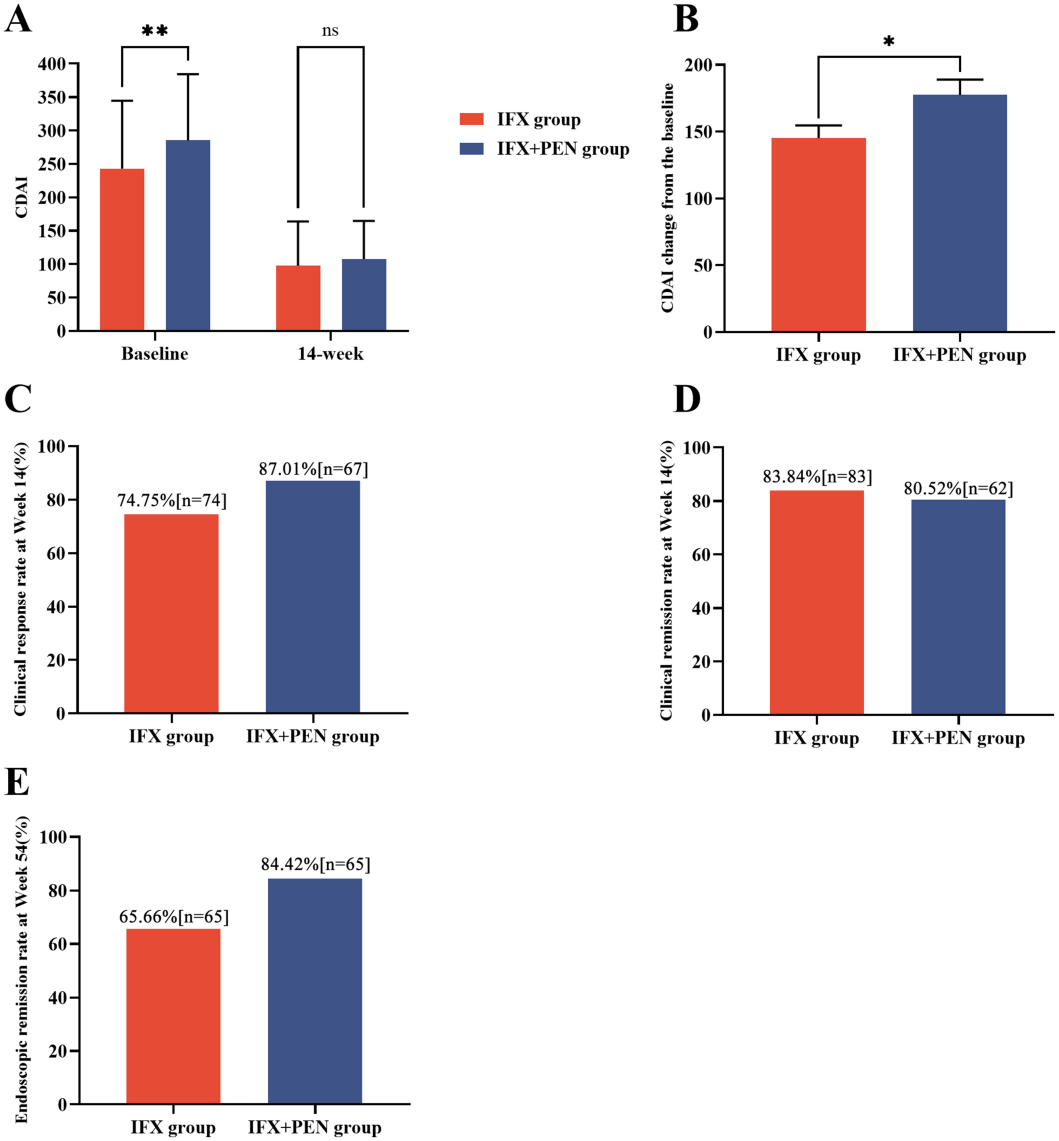
Efficacy between the IFX + PEN group and IFX monotherapy group. **(A)** CDAI scores at baseline and at week 14 in two groups. **(B)** CDAI score change during 14-week period in two groups. **(C)** The number and partition of CD patients achieving clinical response at week 14. **(D)** The number and partition of CD patients achieving clinical remission at week 14. **(E)** The number and partition of CD patients achieving endoscopic remission at week 54.

A significant reduction in the CDAI score was observed in both groups during the initial 14-week follow-up period. At baseline, the CDAI scores in the IFX + PEN group were significantly higher than those in the IFX monotherapy group (285.34 ± 98.80 vs. 242.95 ± 101.49, *p* = 0.006). However, no significant difference was observed at week 14 (107.9 ± 56.90 vs. 97.9 ± 66.10, *p* = 0.075). Notably, the change in CDAI score (ΔCDAI) within the initial 14 weeks was significantly greater in the IFX + PEN group compared to the IFX monotherapy group (177.6 ± 99.57 vs. 145.0 ± 93.94, *p* = 0.026) (Figures 1A,B).

Based on the CDAI score and ΔCDAI, we further calculated the proportion of patients achieving clinical response or clinical remission at week 14. A significantly higher proportion of patients in the IFX + PEN group achieved clinical response compared to the IFX monotherapy group (87.01% vs. 74.75%, *p* = 0.043) (Figure 1C). However, no significant difference was observed in the clinical remission rates between the two groups (Figure 1D). Additionally, a higher proportion of patients in the IFX + PEN group maintained endoscopic remission compared to the IFX monotherapy group at week 54 (84.42% vs. 65.66%, *p* = 0.005) (Figure 1E). Detailed statistical results are provided in Table 2.

**Table 2:** CDAI Scores and efficacy evaluation at week 14 and week 54 between the IFX group and the IFX + PEN group.

Variable	IFX group (*n* = 99)	IFX + PEN group (*n* = 77)	*p*-value
CDAI score at baseline	242.95 ± 101.49	285.34 ± 98.80	0.006
CDAI score at week 14	97.9 ± 66.10	107.9 ± 56.90	0.075
CDAI score change during week 14	145 ± 93.94	177.6 ± 99.57	0.026
Clinical response at week 14, *n* (%)	74 (74.75%)	67 (87.01%)	0.043
Clinical remission at week 14, *n* (%)	83 (83.84%)	62 (80.52%)	0.566
Endoscopic remission at week 54, *n* (%)	65 (65.66%)	65 (84.42%)	0.005

### 3.3 Predictors of endoscopic remission in patients

Univariate logistic regression analysis of the population completing regular follow-up identified male sex and combination therapy with PEN as significant predictors of endoscopic remission at week 54. Meanwhile, a history of CD-related surgery and disease location involving both the colon and ileum were associated with an increased likelihood of endoscopic remission at week 54 (Table 3). Further detailed results are provided in Supplementary Table 1.

**Table 3:** Univariate and multivariable logistic regression analyses for the effect of combination therapy with PEN on endoscopic remission at 54 weeks.

Variable	Non-adjusted model		Multivariate-adjusted Model 1		Multivariate-adjusted Model 2	
	OR (95%CI)	*p*-value	OR (95%CI)	*p*-value	OR (95%CI)	*p*-value
Age	0.98 (0.95, 1.02)	0.2897				
Sex						
Female	1.00					
Male	2.48 (1.17, 5.28)	0.0184				
BMI	0.95 (0.84, 1.06)	0.3604				
Disease location						
L1 (ileal)	1.00		1.00			
L2 (colonic)	0.66 (0.25, 1.71)	0.3890	0.57 (0.19, 1.75)	0.3259		
L3 (ileocolonic)	1.84 (0.81, 4.16)	0.1441	1.80 (0.70, 4.61)	0.2197		
Behavior						
Structuring and penetrating	1.00		1.00			
B1 (non-structuring and penetrating)	0.87 (0.09, 8.14)	0.9028	2.40 (0.19, 30.16)	0.4971		
B2 (structuring)	0.43 (0.04, 4.13)	0.4622	1.16 (0.09, 14.55)	0.9069		
B3 (penetrating)	0.83 (0.07, 10.60)	0.8882	2.03 (0.13, 31.77)	0.6143		
CD-related surgical history	2.45 (0.69, 8.71)	0.1652	3.01 (0.66, 13.69)	0.1544		
IFX	1.00		1.00		1.00	
IFX + PEN	2.83 (1.35, 5.95)	0.0060	3.34 (1.49, 7.50)	0.0034	3.33 (1.39, 7.97)	0.0069

BMI, body mass index; CD, Crohn’s disease; IFX, infliximab; PEN, partial enteral nutrition. Non-adjusted model adjust for: None. Multivariate-Adjusted Model 1 adjust for: Age; Sex; BMI. Multivariate-Adjusted Model 2 adjust for: Age; Sex; BMI; Location; Behavior; CD-related surgical history.

Covariates for multivariable logistic regression analysis were selected based on commonly used clinical indicators and univariate analysis results. Variables included age, BMI, disease location, disease behavior, CD-related surgical history, and treatment with PEN. Notably, after adjusting for potential confounders, combination therapy with PEN emerged as an independent protective predictor of endoscopic remission at 54 weeks in Multivariate-Adjusted Model 1 [OR: 3.34 (95% CI: 1.49–7.50), *p* = 0.0034] and Multivariate-Adjusted Model 2 [OR: 3.33 (95% CI: 1.39–7.97), *p* = 0.0069] (Table 3).

### 3.4 Stratified analysis and interaction analysis

**Figure 2:**
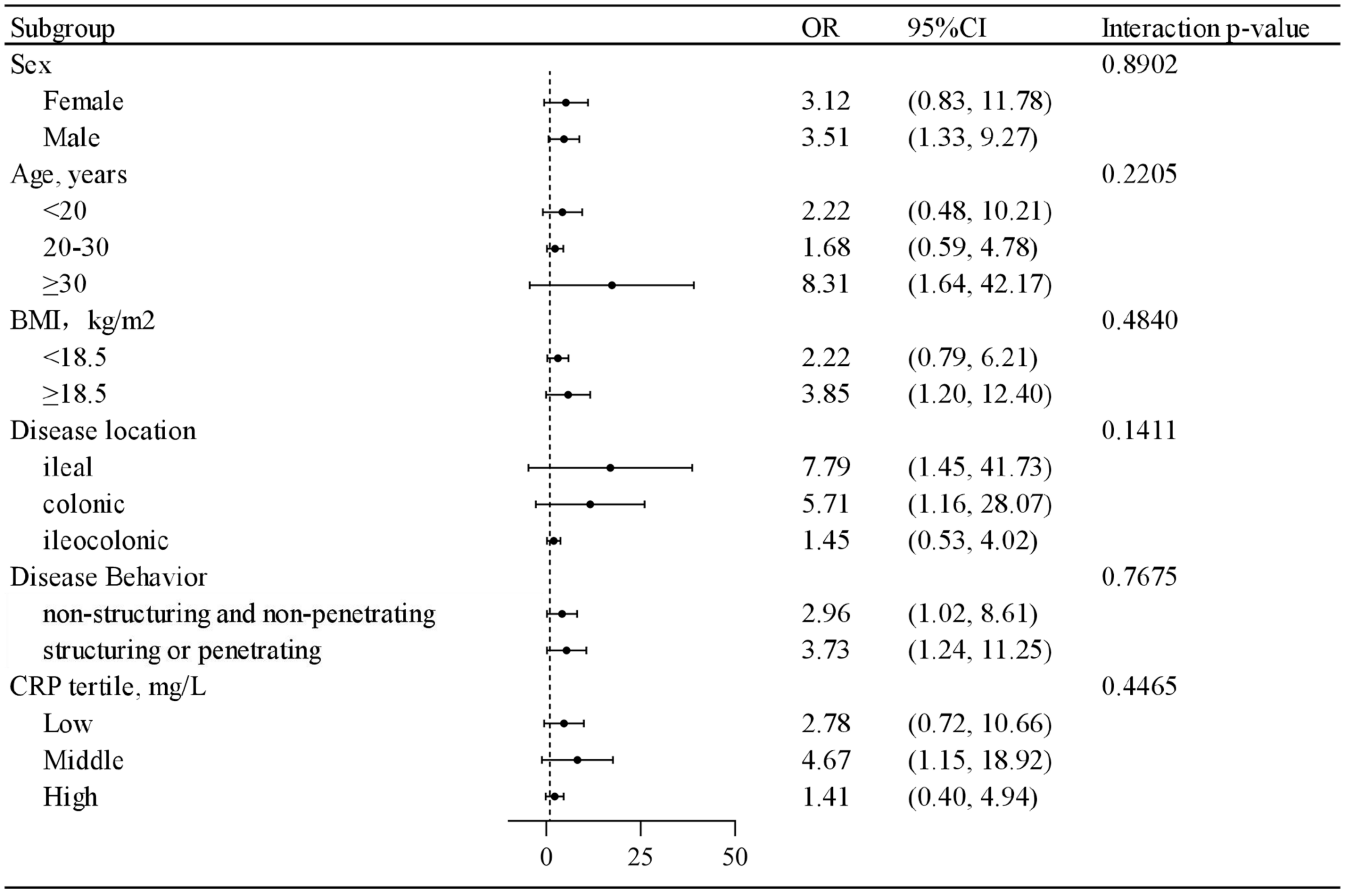
The protective role of treatment combined partial enteral nutrition in achieving endoscopic remission in patients with Crohn’s disease.

This study also presents the number and proportion of cases achieving endoscopic remission across treatment with PEN, stratified by sex, age, BMI, disease location, disease behavior, and CRP level. A positive association between treatment with IFX combined with PEN and endoscopic remission at week 54 was observed across nearly all strata. Furthermore, interaction analysis revealed no significant interactions between PEN and the evaluated potential risk factors (*p* > 0.05 for all interactions) (Figure 2). Detailed results are provided in Table 4.

**Table 4:** Association between combination therapy with PEN and CD activity at week 54 according to baseline characteristics.

Variable	IFX		IFX + PEN		Effect of IFX + PEN		*p*-value for Interaction
	*N*	Remission, *n* (%)	*N*	Remission, *n* (%)	OR (95%CI)	*p*-value	
Sex							0.8902
Female	18	8 (12.31%)	21	15 (23.08%)	3.12 (0.83, 11.78)	0.0924	
Male	81	57 (87.69%)	56	50 (76.92%)	3.51 (1.33, 9.27)	0.0114	
Age (years)							0.2205
Age<20	24	18 (27.69%)	23	20 (30.77%)	2.22 (0.48, 10.21)	0.3048	
20 ≤ Age <30	45	31 (47.69%)	33	26 (40.00%)	1.68 (0.59, 4.78)	0.3326	
Age≥30	30	16 (24.62%)	21	19 (29.23%)	8.31 (1.64, 42.17)	0.0106	
BMI (kg/m^2^)							0.4840
<18.5	37	25 (38.46%)	45	37 (56.92%)	2.22 (0.79, 6.21)	0.1286	
≥18.5	62	40 (61.54%)	32	28 (43.08%)	3.85 (1.20, 12.40)	0.0239	
Disease location							0.1411
L1 (ileal)	23	12 (18.46%)	19	17 (26.15%)	7.79 (1.45, 41.73)	0.0165	
L2 (colonic)	17	7 (10.77%)	15	12 (18.46%)	5.71 (1.16, 28.07)	0.0319	
L3 (ileocolonic)	59	46 (70.77%)	43	36 (55.38%)	1.45 (0.53, 4.02)	0.4712	
Disease behavior							0.7675
Non-structuring and non-penetrating	70	50 (71.43%)	42	37 (88.10%)	2.96 (1.02, 8.61)	0.0465	
Structuring or penetrating	29	15 (51.72%)	35	28 (80.00%)	3.73 (1.24, 11.25)	0.0192	
CRP tertile							0.4465
Low	26	18 (30.00%)	29	25 (40.32%)	2.78 (0.72, 10.66)	0.1364	
Middle	33	19 (31.67%)	22	19 (30.65%)	4.67 (1.15, 18.92)	0.0310	
High	32	23 (38.33%)	23	18 (29.03%)	1.41 (0.40, 4.94)	0.5926	

BMI, body mass index; CRP, C-reactive protein.

### 3.5 Safety

In this study, we evaluated medication-related adverse events occurring during the 54-week follow-up period. As shown in Table 5, no significant differences were observed between the IFX monotherapy group and the IFX + PEN group.

**Table 5:** Incidence of treatment-emergent adverse events during 54 weeks.

Variable	Total (*n* = 176)	IFX group (*n* = 99)	IFX + PEN group (*n* = 77)	*p*-value
Serious infection, *n* (%)	16 (9.09%)	8 (8.08%)	8 (10.39%)	0.597
Infusion related reaction/hypersensitivity, *n* (%)	7 (3.98%)	2 (2.02%)	5 (6.49%)	0.132
Hematologic condition	12 (6.82%)	6 (6.06%)	6 (7.79%)	0.651
Hepatic insufficiency	27 (15.34%)	11 (11.11%)	16 (20.78%)	0.077
Congestive heart failure	0	0	0	-
Demyelinating neurological disorder	0	0	0	-
CD-related unexpected hospitalization	6 (3.41%)	3 (3.03%)	3 (3.90%)	0.754

## 4 Discussion

The clinical application of biologics, represented by IFX, has brought significant benefits to an increasing number of CD patients. However, issues such as loss of response or efficacy to biologic therapy have limited their broader application in clinical practice. Addressing these challenges holds significant clinical importance. This study is aimed at investigating the efficacy of combination therapy with PEN in CD patients. The main findings are outlined below: (1) In induction therapy, combination therapy with PEN can effectively increased the rate of clinical response. (2) In remission-maintaining therapy, combination therapy with PEN is good for endoscopic remission for a long time. (3) Combination therapy with PEN has been identified as an independent protective factor for maintaining endoscopic remission in CD patients.

A newly systematic review involving 20 RCTs for induction therapy pointed out that one-third of CD patients do not respond to initial treatment with TNF antagonists, and another one-third have only a transient response requiring a dose increase or switching to another therapy (22). Clinical response or remission was regarded as an important short-term and intermediate treatment goals in CD patients and those who failed to achieve those goal are recommended to change strategy (3, 23). In this study, we observed that the average CDAI scores between the IFX + PEN group and the IFX group at 14 weeks has no significant difference (107.9 ± 56.90 vs. 97.9 ± 66.10, *p* = 0.075). However, the ΔCDAI score in the IFX + PEN group was significantly higher than that in the IFX group (177.6 ± 99.57 vs. 145.0 ± 93.94, *p* = 0.026). Meanwhile, 67 (87.01%) patients in the IFX + PEN group achieved clinical response compared to 74 (74.75%) in the IFX group (*p* = 0.043), with no significant difference in the rate of clinical remission (83 (83.84%) vs. 62 (80.52%), *p* = 0.566). As shown in the above results, concomitant nutritional therapy can significantly reduce CDAI scores and assist in achieving clinical response during the induction period.

Zhou et al. (24) conducted a 12-week prospective study involving 56 CD patients treated with adalimumab (ADA). They found that the CDAI scores in the combination (ADA + EN) group decreased significantly after 12 weeks of treatment (346.50 ± 124.00 vs. 149.61 ± 76.36, *p* < 0.001), while those in the ADA monotherapy group decreased less (319.90 ± 101.20 vs. 208.73 ± 94.07, *p* = 0.0014). Hisamatsu et al. (25) conducted a 16-week prospective study involving 20 CD patients who did not respond to IFX at a normal dose. They found combination therapy (IFX + EN) significantly increased the rate of clinical response (ΔCDAI > 50-point decrease) and clinical remission compared with IFX monotherapy after dose escalation (both 72.73% vs. 0%, *p* = 0.0256). Similar outcomes were also observed in another 24-week study (26). A retrospective study by Tanaka et al. (27) involved 110 CD biologic-naive adult patients. Within this cohort, 51 (46.4%) CD patients received concomitant elemental diet therapy. Compared to infliximab monotherapy, concomitant elemental diet therapy demonstrated a significantly higher response rate in the inflammatory subgroup (OR = 4.5; 95% CI: 1.7–11.9; *p* = 0.0023), but this phenomenon was not observed in the fistulizing subgroup. Their findings are consistent with our results. However, Matsumoto’s study (28) pointed out that there was no significant difference in the rate of clinical response between groups with or without combined nutritional therapy, which could be mainly attributed to the small number of enrolled participants and the follow-up period being only 2 weeks.

The improvement of clinical symptoms in CD patients does not completely align with the resolution of intestinal mucosal inflammation (29), so endoscopy is considered the gold standard for assessing CD activity. The latest consensus suggests that endoscopic remission (defined as SES-CD < 3 with no ulcer) should be a long-term goal in the treatment of CD, as it is closely related to long-term outcomes (3). Therefore, endoscopic remission at 54 weeks was set as the primary endpoint in this study, and to our knowledge, this is the first study of its kind to use endoscopic remission as the study endpoint.

A retrospective cohort study conducted by Sazuka et al. (30) enrolled 74 adult CD patients who achieved successful induction of clinical remission with IFX induction therapy. They discovered that the cumulative number of patients with LOR to IFX in the combination therapy group was significantly lower than that in the IFX monotherapy group (20.6% vs. 52.3%; OR = 0.23; 95% CI: 0.072–0.64; *p* = 0.0043). Moroi et al. (31) conducted a single-center, retrospective study involving 184 biologic-naive CD patients treated with anti-TNF-*α* antibodies, and they also found that concomitant elemental diet therapy (>900 kcal/day) had a positive influence on biologics retention (HR = 3.19; 95% CI: 1.02–13.89; *p* = 0.0454). In our study, patients in the IFX + PEN group had a higher rate of sustaining remission than those in the IFX monotherapy group at 54 weeks (84.42% vs. 65.66%, *p* = 0.005), and concomitant nutritional therapy was also identified as an independent protective predictor of endoscopic remission. Although the definitions in different studies were not entirely consistent, which was mainly constrained by the limited understanding of CD at the time, it is noteworthy that the above studies have all demonstrated the positive effects of nutritional therapy in IFX treatment. However, several studies present opposing views. A study by Sugita et al. (32) discovered that the cumulative non-ADA-LOR rate between the ED group and the non-ED group showed no difference in Kaplan–Meier analysis among biologic-naive patients. The same phenomenon was also observed in a prospective clinical trial by Yamamoto et al. (33). Nevertheless, we still noted a trend where the proportion of patients maintaining remission in the ED group was higher in their research. This may be attributed to the limited number of participants, the short follow-up period, and the actual adherence to enteral nutrition by patients in the combined group. Currently, the mechanism by which combined nutritional therapy enhances the efficacy of biologics remains unclear. An increasing number of studies support the additive effect of EN itself, such as reducing antigen exposure in intestinal tissues, trace elements aiding mucous repair, nutritional therapy having intrinsic anti-inflammatory effects and so on (21). In Naoko Sugita’s study (32), they found that the level of ADA showed no significant differences at both 28 weeks and 52 weeks, regardless of whether nutritional therapy was administered. However, the level of TNF-ɑ significantly decreased in the combination therapy group compared to the monotherapy group at both 28 weeks and 52 weeks, further supporting the additive effect of EN.

This study systematically evaluated the clinical potential of PEN as an active combination therapy strategy instead of traditional nutritional support in patients receiving IFX treatment. The enrolled patients were representative of clinical practice, with a wide distribution of age, disease duration, disease activity severity, and detailed records of biologics use. Compared to previous studies, this study adopted endoscopic remission—a more stringent and precise standard—to assess CD activity. To minimize the influence of other variables directly or indirectly affecting the research results, we applied appropriate statistical methods, combined with clinical experience, to screen relevant influencing variables and constructed two multivariate models with ORs of 3.34 and 3.33, respectively (both *p* < 0.05). Subgroup analysis and interaction tests further explored the effect of nutrition therapy on patients with different characteristics, and the results strongly support that all CD patients can benefit from nutritional therapy. This is another highlight of this study. The incidence of adverse events within 54 weeks did not show significant differences between the two groups.

This study also has some limitations. As a non-randomized, observational study in a single center, the small patient population size and selection bias are inevitable and can influence the outcome. Besides, strict adherence to the prescribed volume of enteral formula is critical in the follow-up period. As a retrospective study, we can only obtain relevant information according to inpatient or outpatient medical records, which may lead to the outcome being influenced by patient compliance. Combination immunosuppression has been shown to significantly improve clinical outcomes in CD patients treated with IFX. However, only 44 patients received concomitant immunomodulators in this study, potentially limiting the statistical power and introducing bias. Therefore, further validation through larger-scale, rigorously designed studies is warranted. The mechanisms of concomitant nutritional therapy increase the efficacy of IFX are still unclear, but all above studies did not test the concentration of anti-drug antibodies and the trough value of serum biologic concentration, which deserves further research in the future.

## 5 Conclusion

In conclusion, enteral nutrition therapy was an independent protective factor for mucous remission. Enteral nutrition therapy in combination with IFX therapy not only can help to increase the rate of clinical response in induction therapy, but also can prevent the incidence of mucous relapse in the long time.

## Data Availability

The raw data supporting the conclusions of this article will be made available by the authors, without undue reservation.

## Footnotes

^1^ http://www.empowerstats.com

## References

[1] Dolinger M Colombel JF Abreu MT. Crohn's disease. Lancet. (2024) 403:1177–91. doi: 10.1016/S0140-6736(23)02586-2 38437854

[2] Ng SC Shi HY Hamidi N Underwood FE Tang W Benchimol EI. Worldwide incidence and prevalence of inflammatory bowel disease in the 21st century: a systematic review of population-based studies. Lancet. (2017) 390:2769–78. doi: 10.1016/S0140-6736(17)32448-0, PMID: 29050646

[3] Turner D Ricciuto A Lewis A D'Amico F Dhaliwal J Griffiths AM. STRIDE-II: an update on the selecting therapeutic targets in inflammatory bowel disease (STRIDE) initiative of the International Organization for the Study of IBD (IOIBD): determining therapeutic goals for treat-to-target strategies in IBD. Gastroenterology. (2021) 160:1570–83. doi: 10.1053/j.gastro.2020.12.031, PMID: 33359090

[4] Armuzzi A De Pascalis B Fedeli P De Vincentis F Gasbarrini A. Infliximab in Crohn's disease: early and long-term treatment. Dig Liver Dis. (2008) 40:S271–9. doi: 10.1016/S1590-8658(08)60537-X 18599000

[5] Hazlewood GS Rezaie A Borman M Panaccione R Ghosh S Seow CH. Comparative effectiveness of immunosuppressants and biologics for inducing and maintaining remission in Crohn's disease: a network meta-analysis. Gastroenterology. (2015) 148:344–354.e5. doi: 10.1053/j.gastro.2014.10.011, PMID: 25448924

[6] Gordon H Burisch J Ellul P Minozzi S Kopylov U Verstockt B. Ecco guidelines on therapeutics in Crohn's disease: medical treatment. J Crohns Colitis. (2024) 18:1531–55. doi: 10.1093/ecco-jcc/jjae091, PMID: 38877997

[7] Inflammatory Bowel Disease Group, Chinese Society of Gastroenterology, Chinese Medical Association, Inflammatory Bowel Disease Quality Control Center of China. 2023 Chinese national clinical practice guideline on diagnosis and management of Crohn's disease. Chin Med J. (2024) 137:1647–50. doi: 10.1097/CM9.0000000000003222, PMID: 38955665 PMC11268814

[8] Kennedy NA Heap GA Green HD Hamilton B Bewshea C Walker GJ. Predictors of anti-TNF treatment failure in anti-TNF-naive patients with active luminal Crohn's disease: a prospective, multicentre, cohort study. Lancet Gastroenterol Hepatol. (2019) 4:341–53. doi: 10.1016/S2468-1253(19)30012-3, PMID: 30824404

[9] Kumar P Vuyyuru SK Das P Kante B Ranjan MK Thomas DM. Serum albumin is the strongest predictor of anti-tumor necrosis factor nonresponse in inflammatory bowel disease in resource-constrained regions lacking therapeutic drug monitoring. Intest Res. (2023) 21:460–70. doi: 10.5217/ir.2022.00128 36926698 PMC10626021

[10] Roda G Jharap B Neeraj N Colombel JF. Loss of response to anti-TNFs: definition, epidemiology, and management. Clin Transl Gastroenterol. (2016) 7:e135. doi: 10.1038/ctg.2015.63, PMID: 26741065 PMC4737871

[11] Gisbert JP Panés J. Loss of response and requirement of infliximab dose intensification in Crohn's disease: a review. Am J Gastroenterol. (2009) 104:760–7. doi: 10.1038/ajg.2008.88, PMID: 19174781

[12] Qiu Y Mao R Chen BL Zhang SH Guo J He Y. Effects of combination therapy with Immunomodulators on trough levels and antibodies against tumor necrosis factor antagonists in patients with inflammatory bowel disease: a Meta-analysis. Clin Gastroenterol Hepatol. (2017) 15:1359–1372.e6. doi: 10.1016/j.cgh.2017.02.005, PMID: 28232073

[13] Kotlyar DS Osterman MT Diamond RH Porter D Blonski WC Wasik M. A systematic review of factors that contribute to hepatosplenic T-cell lymphoma in patients with inflammatory bowel disease. Clin Gastroenterol Hepatol. (2011) 9:e1:36–41. doi: 10.1016/j.cgh.2010.09.016 20888436

[14] Park KT Colletti RB Rubin DT Sharma BK Thompson A Krueger A. Health insurance paid costs and drivers of costs for patients with Crohn's disease in the United States. Am J Gastroenterol. (2016) 111:15–23. doi: 10.1038/ajg.2015.207, PMID: 26195179

[15] Ding NS Hart A De Cruz P. Systematic review: predicting and optimising response to anti-TNF therapy in Crohn's disease - algorithm for practical management. Aliment Pharmacol Ther. (2016) 43:30–51. doi: 10.1111/apt.13445, PMID: 26515897

[16] Grover Z Lewindon P. Two-year outcomes after exclusive enteral nutrition induction are superior to corticosteroids in pediatric Crohn's disease treated early with Thiopurines. Dig Dis Sci. (2015) 60:3069–74. doi: 10.1007/s10620-015-3722-9, PMID: 26038093

[17] Van Rheenen PF Aloi M Assa A Bronsky J Escher JC Fagerberg UL. The medical management of paediatric Crohn's disease: an ECCO-ESPGHAN guideline update. J Crohns Colitis. (2021) 14:jjaa161. doi: 10.1093/ecco-jcc/jjaa161 33026087

[18] Swaminath A Feathers A Ananthakrishnan AN Falzon L Li Ferry S. Systematic review with meta-analysis: enteral nutrition therapy for the induction of remission in paediatric Crohn's disease. Aliment Pharmacol Ther. (2017) 46:645–56. doi: 10.1111/apt.14253, PMID: 28815649 PMC5798240

[19] Gold SL Steinlauf AF Colombel JF. High prevalence of malnutrition and micronutrient deficiencies in patients with inflammatory bowel disease early in disease course. Inflamm Bowel Dis. (2023) 29:423–9. doi: 10.1093/ibd/izac102 35590456 PMC9977243

[20] Takaoka A Sasaki M Nakanishi N Kurihara M Ohi A Bamba S. Nutritional screening and clinical outcome in hospitalized patients with Crohn's disease. Ann Nutr Metab. (2017) 71:266–72. doi: 10.1159/000485637 29241167

[21] Hirai F Takeda T Takada Y Kishi M Beppu T Takatsu N. Efficacy of enteral nutrition in patients with Crohn's disease on maintenance anti-TNF-alpha antibody therapy: a meta-analysis. J Gastroenterol. (2020) 55:133–41. doi: 10.1007/s00535-019-01634-1 31641874 PMC6981109

[22] Su H Li X Chen Y. Systematic review and bayesian network meta-analysis: comparative efficacy and safety of six commonly used biologic therapies for moderate-to-severe Crohn's disease. Front Pharmacol. (2025) 15:1475222. doi: 10.3389/fphar.2024.1475222 39911832 PMC11794990

[23] Buisson A Gonzalez F Poullenot F. Comparative acceptability and perceived clinical utility of monitoring tools: a nationwide survey of patients with inflammatory bowel disease. Inflamm Bowel Dis. (2017) 23:1425–33. doi: 10.1097/MIB.0000000000001140, PMID: 28570431

[24] Zhou S Li X Chen Y. Prospective study of an adalimumab combined with partial enteral nutrition in the induction period of Crohn's disease. Inflamm Res. (2024) 73:199–209. doi: 10.1007/s00011-023-01828-7 38168701 PMC10824800

[25] Hisamatsu T Kunisaki R Nakamura S Tsujikawa T Hirai F Nakase H. Effect of elemental diet combined with infliximab dose escalation in patients with Crohn's disease with loss of response to infliximab: CERISIER trial. Intest Res. (2018) 16:494–8. doi: 10.5217/ir.2018.16.3.494 30090050 PMC6077314

[26] Nardone OM Calabrese G Testa A. Effectiveness of partial enteral nutrition as add-on to biologics in patients with refractory and difficult-to-treat Crohn's disease: a pilot study. Crohn's Colitis. (2024) 6:otae011. doi: 10.1093/crocol/otae011, PMID: 38464346 PMC10923207

[27] Tanaka T Takahama K Kimura T Mizuno T Nagasaka M Iwata K. Effect of concurrent elemental diet on infliximab treatment for Crohn's disease. J Gastroenterol Hepatol. (2006) 21:1143–9. doi: 10.1111/j.1440-1746.2006.04317.x, PMID: 16824066

[28] Matsumoto T Iida M Kuroki F. Therapeutic efficacy of infliximab on active Crohn's disease under nutritional therapy. Scand J Gastroenterol. (2005) 40:1423–30. doi: 10.1080/00365520510023639 16316890

[29] Laterza L Pizzoferrato M Caioni G. Multiparametric evaluation predicts different mid-term outcomes in Crohn's disease. Dig Dis. (2018) 36:184–93. doi: 10.1159/000487589 29514146

[30] Sazuka S Katsuno T Nakagawa T. Concomitant use of enteral nutrition therapy is associated with sustained response to infliximab in patients with Crohn's disease. Eur J Clin Nutr. (2012) 66:1219–23. doi: 10.1038/ejcn.2012.120, PMID: 23010687

[31] Moroi R Shiga H Kakuta Y. Long-term prognosis of Japanese patients with biologic-naïve Crohn's disease treated with anti-tumor necrosis factor-α antibodies. Intest Res. (2019) 17:94–106. doi: 10.5217/ir.2018.00048 30508475 PMC6361023

[32] Sugita N Kato S Ohmiya N. Efficacy of a concomitant elemental diet to reduce the loss of response to adalimumab in patients with intractable Crohn's disease. J Gastroenterol Hepatol. (2018) 33:631–7. doi: 10.1111/jgh.13969 28857255

[33] Yamamoto T Nakahigashi M Umegae S. Prospective clinical trial: enteral nutrition during maintenance infliximab in Crohn's disease. J Gastroenterol. (2010) 45:24–9. doi: 10.1007/s00535-009-0136-5, PMID: 19798465

