# Supplementary material for: The efficacy of infliximab combined with partial enteral nutrition in the treatment of Crohn’s disease: a cohort study
Source: Front Nutr. 2025 Jun 18;12:1591954. doi: 10.3389/fnut.2025.1591954 (PMC12218248; doi:10.3389/fnut.2025.1591954)
Supplement: Supplementary file 1 [file Table_1.docx]

Supplementary Material

Supplementary Table 1: Univariate Regression for the Effect of Combination Therapy with PEN on Endoscopic Remission at Week 54

| Variable | Statistics | OR (95%CI) | P-value |
| --- | --- | --- | --- |
| Age (years) | 26.38 ± 9.34 | 0.98 (0.95, 1.02) | 0.2897 |
| Disease duration (months) | 2.00 (1.00-28.75) | 1.00 (0.99, 1.01) | 0.9918 |
| BMI (kg/m^2^) | 19.23 ± 2.83 | 0.95 (0.84, 1.06) | 0.3604 |
| CDAI score | 261.50 ± 102.24 | 1.00 (1.00, 1.00) | 0.7510 |
| SES-CD score | 9.00 (6.00-15.00) | 0.99 (0.95, 1.04) | 0.7097 |
| CRP (mg/L) | 18.00 (7.51-38.40) | 1.00 (0.99, 1.01) | 0.8907 |
| ALB (g/L) | 37.77 ± 5.52 | 1.03 (0.97, 1.10) | 0.2931 |
| Sex |  |  |  |
| Female, n(%) | 39 (22.16%) | 1.0 |  |
| Male, n(%) | 137 (77.84%) | 2.48 (1.17, 5.28) | 0.0184 |
| Age at diagnosis, n(%) |  |  |  |
| A1(≤16 years), n (%) | 23 (13.07%) | 1.0 |  |
| A2(17-40 years), n (%) | 140 (79.55%) | 1.06 (0.39, 2.90) | 0.9114 |
| A3(＞40 years), n (%) | 13 (7.39%) | 0.56 (0.13, 2.42) | 0.4412 |
| Disease location |  |  |  |
| L1(ileal), n(%) | 42 (23.86%) | 1.0 |  |
| L2(colonic), n(%) | 32 (18.18%) | 0.66 (0.25, 1.71) | 0.3890 |
| L3(ileocolonic), n(%) | 102 (57.95%) | 1.84 (0.81, 4.16) | 0.1441 |
| L4(Upper digestive tract involved)(yes), n (%) | 16 (9.09%) | 0.56 (0.19, 1.63) | 0.2832 |
| Disease behavior |  |  |  |
| structuring and penetrating, n (%) | 5 (2.84%) | 1.0 |  |
| B1(non-structuring and  Penetrating), n (%) | 112 (63.64%) | 0.87 (0.09, 8.14) | 0.9028 |
| B2(structuring), n (%) | 46 (26.14%) | 0.43 (0.04, 4.13) | 0.4622 |
| B3(penetrating), n (%) | 13 (7.39%) | 0.83 (0.07, 10.60) | 0.8882 |
| perianal disease(yes),n(%) | 82 (46.59%) | 1.34 (0.68, 2.64) | 0.4037 |
| Extra-intestinal manifestations(yes), n (%) | 8 (4.55%) | 0.57 (0.13, 2.50) | 0.4591 |
| Smoking status(yes), n (%) | 14 (7.95%) | 1.32 (0.35, 4.98) | 0.6769 |
| CD-related surgical history(yes), n (%) | 22(12.50%) | 2.45 (0.69, 8.71) | 0.1652 |
| Concomitant immunomodulatory treatment(yes), n (%) | 48 (27.27%) | 0.70 (0.34, 1.46) | 0.3457 |
| Use of biologics for the frst time(yes), n (%) | 149(84.66%) | 1.01 (0.40, 2.58) | 0.9784 |
| IFX+PEN, n (%) | 77(43.75%) | 2.83 (1.35, 5.95) | 0.0060 |

When conducting logistic regression analysis on all binary variables, the event-negative group is used as the baseline.

BMI = body mass index; CDAI score = Crohn’s Disease Activity Index score; SES-CD = Simple Endoscopic Score for Crohn’s Disease; CRP= C-reactive protein; ALB = Albumin; CD = Crohn’s disease; IFX = infliximab; PEN = partial enteral nutrition
